# Implant Surface Variability Between Progressive Knife-Edge Thread Design and International Organization for Standardization Thread with and Without Tapping Area: A Model Analysis

**DOI:** 10.3390/ma18225113

**Published:** 2025-11-11

**Authors:** Davide Farronato, Luca Poncia, Marco Vidotto, Vittorio Maurino, Leonardo Romano

**Affiliations:** 1 Department of Medicine and Technological Innovation, School of Dentistry, University of Insubria, 21100 Varese, Italy; davide@farronato.it; 2 Department of Medicine and Surgery, School of Dentistry, University of Insubria, 21100 Varese, Italy; lponcia@studenti.uninsubria.it; 3 Prodent Italia S.r.l., 20016 Pero, Italy; prg@prodentitalia.it; 4 Department of Biotechnology and Life Sciences, School of Dentistry, University of Insubria, 21100 Varese, Italy; vittorio.maurino@uninsubria.it

**Keywords:** implantology, biomechanics, thread design, knife-edge thread, ISO thread, surface area

## Abstract

An implant’s thread design plays a key role in enhancing primary stability by optimising the distribution of loading forces and biomechanical structural interlocking. An increase in bone-to-implant contact (BIC) surface availability affects osseointegration timing and leads to different biomechanical behaviours. To assess their theoretical impacts on osseointegration functionality, this study aims to analyse and compare the surface areas of two different thread designs: progressive knife-edge and V-shaped metric ISO ones. Six implant models are virtually created, with progressive knife-edge threads, non-self-tapping ISO threads, and ISO threads with tapping areas, considering two arbitrary diameters (3.8 mm and 4.6 mm). For both diameters, the models also have identical lengths (9.5 mm) and external outlines. The total, superior half, and inferior half external surface areas are measured using a digital tool (SolidWorks 2023 SP 5.0, Dassault Systèmes, Waltham, MA, USA). Then, the percentage difference in external surface area (ΔESA) is calculated. A greater ΔESA is found in the knife-edge design compared to the ISO thread self-tapping implants for the 4.6 mm diameter (ΔESA = +9.9%). However, for the 3.8 mm diameter, the ΔESA is −1.5% in favour of the ISO self-tapping model. Considering the apical half of the models, the ΔESA is always greater in the knife-edge models, varying from +9.3% to +23.5%. Implants with progressive knife-edge threads offer a significantly larger external surface area than those with ISO threads for the 4.6 mm rather than the 3.8 mm diameter. Considering the apical halves of the implants, the tapping area negatively affects the ΔESA, as well as the ISO thread design. Future research is needed to investigate whether the inspected surface area differences correspond to significant primary and secondary stability variations.

## 1. Introduction

Implant stability is a condition in which an implant has no clinical mobility [1], and it is divided into primary or mechanical and secondary or biological stability. The former is the stability achieved immediately after implant placement and reflects the degree of mechanical interlocking between the bone and implant [2], while the latter is defined as a structural and functional connection between the living bone and implant surface at the microscopic level [3,4]. Secondary stability is also known as osseointegration, and it is measured by the bone-to-implant contact (BIC), which is the percentage of vital bone in direct contact with the implant surface [5]. After implant placement, biological stability gradually replaces mechanical stability through the substitution of peri-implant bone with newly formed bone [6,7]. During this transition, a stability drop is usually found to occur [8].

Among the metrics used to measure implants’ primary stability and control stability over time, the insertion torque (IT) and implant stability quotient (ISQ) are the most reliable and commonly used [9]. However, the relationship between the IT and ISQ is still unclear, and the literature data are conflicting [10].

Primary stability is influenced by the bone quality and quantity, surgical technique, and implant design [11], while secondary stability depends on the primary stability, implant surface topography, and patient-related factors [12,13]. Among the variables that can determine implant stability and subsequent osseointegration, the thread design is a relevant factor [14]. This is because the thread shape influences both the implant surface area and the direction and distribution of occlusal loads to the surrounding bone [15]. In particular, thread designs that increase the implant surface area augment the initial surface contact area between the implant and bone, allowing better primary stability [16] and a higher BIC at the end of the osseointegration process for better secondary stability [17]. According to Kreve et al. [17], this correlation exists, although it must be considered that biological proof of this principle is necessary for any individual design. At the same time, thread shapes that minimise shear forces, transmitting adequate amounts of compressive and tensile forces, promote the achievement and maintenance of osseointegration [18].

The first thread design used in the history of implantology was the V shape or ISO, which was introduced by Branemark and is still widely applied. Afterwards, different thread shapes emerged across the market, including the buttress, reverse buttress, square, and, more recently, the knife-edge one. Alqahtani et al. [19] investigated the influence of the thread design on implant stability by means of finite element analysis (FEA), finding that square thread implants allow better stability and minimal von Mises stress and strains compared to buttress and V-shaped threads. The implant diameter also affected the stability, with a reduction in micromovement, stress, and strain as the diameter increased. Knife-edge threads were not considered.

From the research conducted by the authors, the literature lacks studies comparing the characteristics of knife-edge threads with those of other designs. The reason is that several studies have focused on this thread design without a control group represented by another implant thread design. In particular, two studies can be found, both of which considered V-shaped and knife-edge threads. McCullough et al. [20] evaluated implant stability by means of the ISQ for the first 8 weeks after implant placement, obtaining similar primary stability between the two groups of thread designs (V-shaped and knife edge). The V-shaped thread group showed an ISQ decrease until the fourth week, while the latter group’s ISQ remained almost stable. The absence of a stability drop during the healing phase aligns with the results of Zita Gomes et al. [21] and Hoekstra et al. [22] regarding the same implant thread design. The other study was a comparative analysis of ISO and knife-edge threads led by Mangano et al., with histologic and histomorphometric evaluations performed 8 weeks after implant placement, highlighting the ability of the knife-edge design to increase the BIC [23]. These results could be related to the larger surface areas of implants with knife-edge threads compared to V-shaped thread implants, with the same length and diameter and better load distribution.

The purpose of this model analysis is to evaluate the total surface area of a knife-edge thread implant model compared with two conventional V-shaped thread implant models: one with a self-tapping area and the other non-self-tapping. The designs under comparison have the same diameter, length, and external outline and differ only in the thread design. We arbitrarily choose to inspect two fixture diameters, 3.8 mm and 4.6 mm, and the comparison is executed both at the coronal and apical halves of the implants. This decision is made arbitrarily to underline their different impacts on the implant surface area. The null hypothesis is that the total surface area of the knife-edge group will be equal to or less than that of each ISO thread group, for both diameters, meaning that the knife-edge thread design alone does not augment the total surface area.

## 2. Materials and Methods

**Figure 1:**
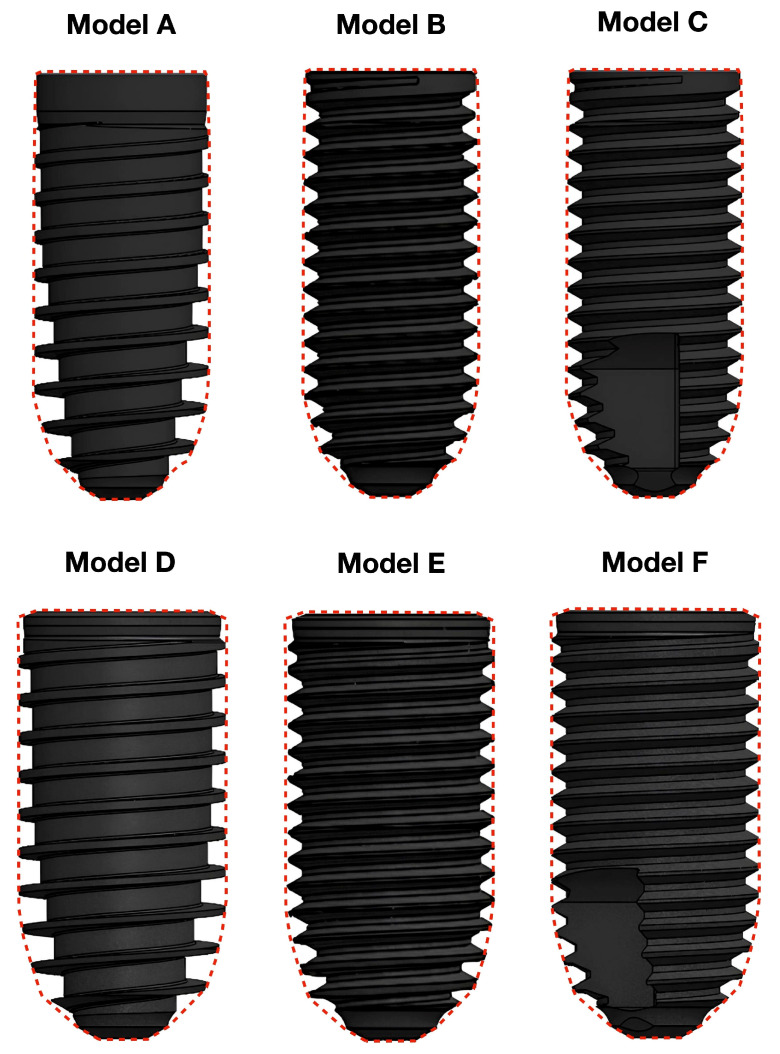
The six types of implants selected for the study. Model A is the knife-edge thread implant with 3.8 mm diameter and 9.5 mm length; model B has identical dimensions but V-shaped threads; model C features V-shaped threads with the addition of a double tapping area in the apical half. Models D, E and F have the same characteristics as models A, B and C, respectively, but a diameter of 4.6 mm. The dotted line shows the external outline of the fixture, which is cylindrical in the coronal portion and conical in the apical one, with a 10° taper angle.

Six implant models are arbitrarily selected (see Figure 1 and Table 1), and all of them are built with SolidWorks (SolidWorks 2023 SP 5.0, Dassault Systèmes, Waltham, MA, USA). The model A implant (Proshape, Prodent Italia, Pero, Italy) is a non-self-tapping screw-shaped implant that presents a cylindrical external outline (the contour determined by the line that joins the tip of the threads), which becomes conical in the apical portion, with a taper angle equal to 10°. The diameter of the fixture is 3.8 mm, while the length is 9.5 mm. The connection is an internal conical one. Regarding the thread design, model A has knife-edge progressive threads, so the thread depth progressively increases in the apical direction, while the implant core diameter reduces. The thread pitch is equal to 0.85 mm.

**Figure 2:**
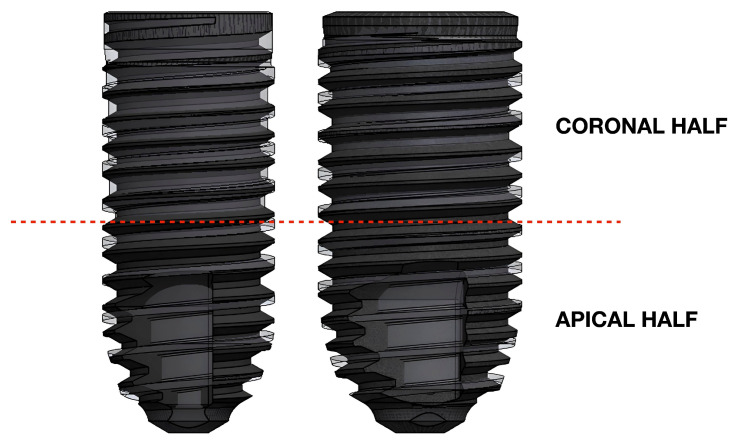
The image shows, on the left and on the right, the superimposition of models A, B, and C and models D, E, and F, respectively. Note that the three implant fixtures on each side have an equal length, diameter, and external outline, while differing in the morphology of the threads and in the presence/absence of the tapping area.

Model B is created according to model A to have the same length, diameter, and external outline, which means that the most external points of the threads of both model A and B share the same profile (see Figure 1 and Figure 2), but it has an ISO (V-shaped) thread design, comparable with the first Branemark implant. The thread pitch is equal to 0.6 mm, while the depth is 0.32 mm. As a consequence of the aforementioned differences between model A and B, and of the requirement to maintain the same outline, the helicoidal profile that the thread follows along the implant is built differently.

A third model (model C), with the same characteristics as model B, is projected by adding a double tapping area in the apical portion, according to the Branemark implant model.

Model D (Proshape, Prodent Italia, Pero, Italy) has the same characteristics as model A but a wider diameter of 4.6 mm. Models E and F are obtained from model D, similarly to the method previously described for models B and C. Therefore, both have the same external outline, diameter, and length as model D but a V-shaped thread design (see Figure 1 and Figure 2); model E does not have a tapping area, while, in model F, one is present.

The implant models selected are considered without any surface treatment that could alter the surface area measures. All surfaces are explicitly checked to ensure proper joining, without discontinuities or voids, eliminating any operator-dependent measurement bias.

For each of the six groups, four parameters are evaluated using a digital tool (SolidWorks 2023 SP 5.0, Dassault Systèmes, Waltham, MA, USA), which performs mathematically precise surface calculations within a fully controlled digital environment. The first parameter is the total surface area, which is defined as the sum of the areas of all external surfaces of an implant, expressed in square millimetres (mm^2^). The second is the external surface area (ESA), which is the area of the outer surfaces of an implant that could contribute to the BIC (i.e., the area of the external surface excluding the connection platform, which is identical in the fixtures with the same diameter). Each implant model is divided into two halves, coronal and apical, by a plane passing through the midpoint of the fixture axis and perpendicular to it. Then, the ESA is also calculated considering the coronal (ESA_COR_) and the apical (ESA_API_) halves separately.

Therefore, the obtained virtual measurements are directly compared with each other through absolute numerical and percentage differences. In particular, the ESA of model A is compared with that of models B and C, while the ESA of model D with that of models E and F. In all cases, the total, coronal half, and apical half ESAs are compared. The percentage external surface difference (ΔESA%) is calculated as the ratio between ΔESA and ESA_ISO model_, where ΔESA is the difference between the ESA of the knife-edge model and the ESA of the ISO model, as shown in Equation (1). When the outcome of the equation is positive, it means that the ESA of the knife-edge model is larger than that of the ISO model by a percentage expressed with respect to the latter, while, when the outcome of the equation is negative, the opposite is true.

(1) $$\;\Delta \mathrm{ESA}\left(\%\right)=\frac{{\mathrm{ESA}}_{\text{knife-edge model}}-{\mathrm{ESA}}_{\mathrm{ISO}\mathrm{model}}}{{\mathrm{ESA}}_{\mathrm{ISO}\mathrm{model}}}\times 100\;.\;$$

## 3. Results

The results obtained are summarised in the table below (Table 2).

Comparing the model A and model B implants, the ΔESA obtained is equal to −5.2% in favour of model B; considering the coronal halves of the two models, the ΔESA is −18.9%, while, for the apical half, it is +9.3% in favour of model A. The ΔESA calculated between model A and model C is −1.5%; it is −18.9% in the coronal half and +18.4% in the apical one.

For implants with a 4.6 mm diameter, the comparison between the model D and model E groups results in a ΔESA of +5.9% in favour of model D, where −2.2% is found for the coronal halves of the implants and +14.3% for the apical. The ΔESA calculated between model D and model F is +9.9%, ΔESA_COR_ is −2.2%, and ΔESA_API_ is +23.5%.

## 4. Discussion

The purpose of this model analysis is to show the effects of two different thread shapes (knife edge and V-shaped) on the total surface area in implants with the same diameter, length, and outline.

Considering the diameter of 3.8 mm, the ΔESA is −5.2% in favour of the ISO thread non-self-tapping model, while, for the diameter of 4.6 mm, it is +5.9% in favour of the knife-edge one. However, most of the commonly used implant fixtures with V-shaped threads have a tapping area.

Comparing the knife-edge thread models with the two created with V-shaped threads and tapping areas, for the 3.8 mm diameter, the ΔESA is −1.5% in favour of the latter, while, for the 4.6 mm diameter, it is +9.9% in favour of the knife-edge thread design, because the tapping area reduces the ESAs of models C and F. Therefore, the knife-edge design with implants that have a 4.6 mm diameter allows a significant gain in ESA compared to the realistic implant models with V-shaped threads.

A more detailed analysis of the data obtained shows that the large gain in the ESA of the knife-edge models is in the apical half. Specifically, for the fixture diameter of 3.8 mm, the ΔESA_API_ is +9.3% and +18.4% when comparing model A with model B and model A with model C, respectively. For the 4.6 mm diameter, the ΔESA_API_ is +14.3% and +23.5% in favour of model D, as compared with models E and F, respectively. This aspect can be explained by the progressive thread design, where the increase in thread depth in the apical portion of the knife-edge implant is related to an increase in the ESA. The difference in the apical half of the fixture is greater when comparing the knife-edge models with the realistic ISO models, because the tapping area further reduces the ESA_API_ in these implants.

These results could have possible clinical consequences. In fact, since the thread morphology is part of the implant macro-design, which has an important influence on the primary stability and osseointegration, it has to be managed in order to guarantee the best result in different clinical conditions [14].

In particular, implants with knife-edge threads may be useful in certain conditions, such as low-density bone, to achieve sufficient stability for immediate loading, i.e., a minimum ISQ of 70 for a single implant [24,25,26], or in the case of using short implants. In these conditions, the advantage of using the knife-edge design rather than the V-shaped one is greater when choosing wide fixture diameters, because the ΔESA increases with augmented diameters. Furthermore, the gain in the ESA_API_ of the progressive knife-edge thread implants may have an important role with a dual advantage. On one hand, it can significantly augment the mechanical anchorage of the implant to the bone and thus the primary stability. In fact, Romanos et al. established that the apical portion of an implant with a progressive thread depth contributes 30–43% of the total primary stability [27]. On the other hand, this type of thread allows a higher ESA in the portion where the implant is in contact with the trabecular bone component, in which bone apposition begins earlier than in the cortical one [28].

However, in the presence of high-density bone, the choice of a thread design that results in a lower ESA could avoid excessive insertion torque (IT) and consequent complications. In fact, an IT above a certain level may cause excessive bone compression, leading to biological complications such as microcracks or bone necrosis. In particular, microfractures may occur when the bone strain generated by the high IT exceeds its elastic properties, while compression necrosis is related to ischemia due to compromised microcirculation [29,30]. A high IT can also result in the deformation or fracture of the implant connection [31]. Furthermore, this condition may prevent the implant from being completely placed subcrestally when the implant’s optimal placement is set below the crest, so that part of the implant’s rough surface remains exposed, with an increased risk of plaque contamination of the treated exposed surface [32], potentially leading to peri-implantitis [33]. Thus, the risks of these possible biological and mechanical complications could be avoided with a correct implant thread design choice by the clinician during surgical planning, while still maintaining appropriate primary stability.

Regarding implant stability, the literature regarding its objective assessment suggests that, among the different possibilities available, the most trustworthy and frequently used metrics are the IT and ISQ [34], but a single superior method cannot be established [35]. Regarding the factors that could influence these parameters, the IT is affected by the bone quality at the implant site, the surgical procedure (shape of osteotomy), and the implant macro-design characteristics [2,36], such as the thread shape [37]. The ISQ is also influenced by the bone density and implant geometry [10], and more variables may be found in future research.

A study by Baldi et al. [38] found a linear relationship between moderate IT values (30–50 N·cm) and the ISQ when using implants with knife-edge threads, while a non-linear relationship was evidenced with IT lower than 30 N·cm or higher than 50 N·cm. For IT values greater than 50 N·cm, there was no significant increase in primary stability with this type of thread. These results align with those of Nevins et al. [39], who used implants positioned in three different conditions (high, moderate, and low compression of peri-implant bone), demonstrating that high compression (i.e., high insertion torque) is not related to higher ISQ values. Since high IT can result in excessive bone compression with higher bone resorption [40] and implant damage [41], maximum torque of 50 N·cm should be ensured with this type of implant thread to reduce the risk of complications [38].

**Figure 3:**
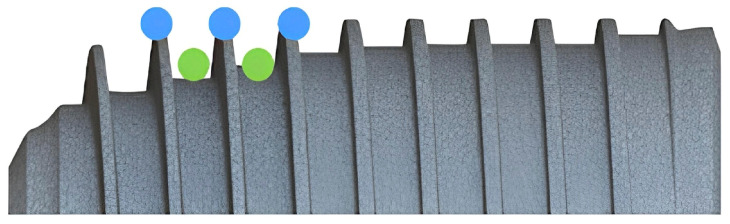
The image shows the model A implant. In blue, the tip of the knife-edge thread is highlighted, while, in green, the areas between the threads are indicated.

A clinical trial using implants with knife-edge threads in three different conditions (non-regenerated, partially regenerated, and nearly totally regenerated bone) showed no stability (ISQ) decrease in the first two months after implant placement [21]. These results are similar to those of McCullough et al. [20] and Hoeckstra et al. [22] regarding the same thread design, where they found similar torque values at implant placement and after 2 weeks, with significantly increased torque-out values after 6 weeks [22]. Therefore, the use of implants with knife-edge threads can also have the advantage of avoiding the lag phase, leading to higher success rates in difficult areas [21]. The reason behind these results could be the fact that knife-edge threads seem to provide a greater surface area for the establishment of primary and secondary stability, especially with wider diameters (see Table 2). On the other hand, this thread design may present a high compression zone at the tip, whose aim is to ensure primary stability, while the zone near the implant core may be a low-compression one, increasing the space for the formation of coagulum and a more efficient neo-angiogenesis process [2], which are the basis for the establishment of secondary stability (Figure 3). This is supported by histometric studies, which have stated that providing a free space between the implant threads and bone, referred to as the healing chamber or clot chamber—for example, by means of longer threads—creates a decompression area that is immediately filled by blood clotting after implant placement, leading to higher percentages of new bone formation [42,43,44].

Overall, this study demonstrates that two implants with an equal diameter and length but different thread shapes can result in a significant difference in surface area with important clinical consequences. In particular, the greater ESA found in the implants with knife-edge threads, especially with a 4.6 mm diameter, compared to those with V-shaped threads, given the same diameter, length, and outline, might lead to higher values of primary stability and subsequently to a more efficient osseointegration process, which aligns with the results obtained by previous studies on this thread design. Therefore, knife-edge threads can be useful to enhance the primary stability in cases of low bone density or to allow immediate loading. On the other hand, in high-density bone, this type of implant might cause excessive torque and the compression of the bone, as well as incomplete implant seating, with biological and technical complications. Another advantage is that implants with knife-edge threads with smaller diameters or lengths have a surface area similar to those with V-shaped threads with greater dimensions. Therefore, they can be used in cases of horizontal or vertical bone volume deficiency to obtain better stability, reducing the need for bone augmentation procedures and preventing damage to noble structures, such as the inferior alveolar nerve or maxillary sinus, by maintaining a safe distance.

As a model analysis, the chosen designs allow a theoretical comparison due to the need for bias reduction. The measurements obtained in the presented analysis are not necessarily definitive because they were calculated based on theoretical models. Real implant fixture calculations may be less precise due to production tolerances; thus, the results shown can only be considered as a theoretical reference. Clinically speaking, the relationship between the implant design and the drill shape or the specific purpose of use involves a balance, determined by the producer, that aligns with specific indications. As a consequence, the aim of this article is strictly technical, and it does not seek to suggest designs but to highlight theoretical advantages that might be considered together with a wide variety of factors when creating a new design or evaluating an existing one.

Further evaluations should be performed in vitro to test the mechanical strength of screws in bone samples with different densities, before in vivo evaluation, to confirm the different clinical outcomes hypothesised based on the implant thread design type. The surface–BIC correlation must be biologically proven with histological measurements; therefore, this correlation for different thread designs can be considered a proof of principle until biological confirmation is obtained. It would also be useful to analyse the impacts of different thread geometries on the bone stress distribution and investigate whether the micro- and macro-design characteristics can influence the relationship between a specific thread design and the related clinical outcomes. Finally, since the diameters and lengths were arbitrarily chosen, future studies could be conducted on a larger sample size in order to better understand the influence of these parameters on the implant surface area in relation to the thread design.

## 5. Conclusions

In this article, knife-edge thread implants are shown to have a wider external surface area available than self-tapping implants with V-shaped threads with the same diameter (4.6 mm), length (9.5 mm), and fixture shape, with an ΔESA of +9.9%. The greater gain in surface area occurs in the apical portion of the knife-edge model (ΔESA_API_ = +23.5). Further studies are needed, using bone samples with standardised densities, to verify whether the surface area differences found correspond to significant IT and ISQ variations.

## Figures and Tables

**Table 1:** The principal characteristics of the models considered in the study.

Implant Model	Diameter (mm)	Length (mm)	Thread Depth (mm)	Thread Angle (°)	Thread Pitch (mm)	Total Surface Area (mm^2^)
Model A	3.8	9.5	NA *	NA *	0.85	156.020
Model B	3.8	9.5	0.32	60°	0.6	164.332
Model C	3.8	9.5	0.32	60°	0.6	158.268
Model D	4.6	9.5	NA *	NA *	0.85	205.466
Model E	4.6	9.5	0.32	60°	0.6	194.425
Model F	4.6	9.5	0.32	60°	0.6	187.584

* In accordance with confidentiality agreements with the manufacturer, some details of the implant design have been intentionally omitted (NA). We have verified all geometric parameters and confirmed the correctness of the measurements obtained.

**Table 2:** A summary of the results obtained.

Implant Model	ESA_COR_ (mm^2^)	ΔESA_COR_	ESA_API_ (mm^2^)	ΔESA_API_	ESA (mm^2^)	ΔESA
Model A	66.936	−18.9%	85.599	+9.3%	152.535	−5.2%
Model B	82.509		78.338		160.847	
Model A	66.936	−18.9%	85.599	+18.4%	152.535	−1.5%
Model C	82.509		72.274		154.783	
Model D	92.961	−2.2%	104.982	+14.3%	197.943	+5.9%
Model E	95.034		91.868		186.902	
Model D	92.961	−2.2%	104.982	+23.5%	197.943	+9.9%
Model F	95.034		85.027		180.061	

## Data Availability

The original contributions presented in this study are included in the article. Further inquiries can be directed to the corresponding author.

## Abbreviations

The following abbreviations are used in this manuscript:

BIC	Bone-to-implant contact
ESA	External surface area
ESA_API_	External surface area of apical half
ESA_COR_	External surface area of coronal half
ΔESA	Percentage of external surface difference
ΔESA_API_	Percentage of external surface difference in apical half
ΔESA_COR_	Percentage of external surface difference in coronal half
FEA	Finite element analysis
ISQ	Implant stability quotient
ISO	International Organization for Standardization
IT	Insertion torque
